# Neurodegeneration Within the Spectrum of Pervasive Developmental Disorders

**DOI:** 10.7759/cureus.99010

**Published:** 2025-12-11

**Authors:** Diana Mihalcea, Marius P Iordache, Claudia Angelescu

**Affiliations:** 1 Psychiatry, Regina Maria Clinic, Bucharest, ROU; 2 Faculty of Medicine, Titu Maiorescu University, Bucharest, ROU; 3 Neurology, Clinical County Emergency Hospital Ilfov, Bucharest, ROU; 4 Psychiatry, Polimed Clinic, Bucharest, ROU

**Keywords:** atypical aging, autism spectrum disorder, fragile x–associated tremor/ataxia syndrome, neurodegeneration, neurodevelopmental disorders, pervasive developmental disorders, rett syndrome

## Abstract

The historical framework of pervasive developmental disorders (PDD) continues to hold relevance despite its replacement by the Neurodevelopmental Disorders category in DSM-5. Emerging evidence indicates that neurodevelopmental and neurodegenerative mechanisms, traditionally considered distinct, may intersect in individuals formerly classified under PDD. Shared biological pathways, including aberrant synaptic pruning, mitochondrial dysfunction, immune dysregulation, and impaired proteostasis, suggest potential long-term neurological vulnerability, particularly in autism spectrum disorder (ASD), the principal diagnosis within the former PDD group. Clinical overlap is most evident in "bridging conditions" such as Rett syndrome, *MECP2*-related disorders, and Fragile X-associated tremor/ataxia syndrome, which illustrate the continuum between developmental and degenerative processes. As individuals with ASD increasingly reach older adulthood, gaps in longitudinal data hinder understanding of risks related to cognitive decline, dementia, and atypical aging. Additionally, chronic stress, psychiatric comorbidities, and sensory dysregulation may further influence neuroinflammatory and neurodegenerative pathways. Recognizing these intersections provides opportunities for early neuroprotective interventions and biomarker-driven risk stratification. This editorial argues for a lifespan-oriented, integrative model that bridges developmental and degenerative neuroscience, aiming to better characterize and support the long-term neurological health of individuals previously encompassed by the PDD construct.

## Editorial

The historical construct of pervasive developmental disorders (PDD) has long framed our understanding of early-onset conditions characterized by social, communication, and behavioural impairments. Although DSM-5 has replaced PDD with the broader category of Neurodevelopmental Disorders, the concept remains clinically and biologically relevant, especially when examining intersections with neurodegeneration [1]. Increasingly, evidence suggests that neurodevelopmental and neurodegenerative pathways, traditionally viewed as distinct, may converge in subtle but meaningful ways. This overlap challenges long-standing assumptions about the trajectory, vulnerability, and long-term outcomes of individuals formerly categorized under PDD.

Autism spectrum disorder (ASD), the central diagnosis within the historical PDD group, is conventionally seen as a stable, lifelong neurodevelopmental condition rather than a progressive one [2]. Yet a growing body of neurobiological research points to abnormalities in synaptic pruning, mitochondrial function, immune signalling, and proteostazis that resemble mechanisms implicated in classical neurodegenerative diseases. While ASD is not considered a neurodegenerative disorder in itself, these shared pathways invite a reconsideration of its long-term neurological vulnerability. For example, chronic neuroinflammation, well described in ASD, may contribute to altered neural connectivity and could predispose individuals to age-related cognitive decline. Similarly, genetic findings involving genes critical for synaptic maintenance, such as *SHANK*, *PTEN*, and *MECP2*, highlight the blurred boundaries between atypical development and neurodegeneration [3].

One of the most compelling clinical overlaps emerges in disorders genetically linked to both neurodevelopmental phenotypes and degenerative trajectories. Rett syndrome and other *MECP2*-related disorders exemplify a neurodevelopmental onset followed by clear neurodegenerative features, including loss of motor and cognitive skills [3]. Fragile X-associated tremor/ataxia syndrome (FXTAS) provides a mirror image: an adult-onset neurodegenerative disease originating from the same molecular background as a childhood neurodevelopmental syndrome [4]. These "bridging conditions" underscore the continuum along which developmental and degenerative mechanisms may interact.

From a clinical standpoint, the possibility of neurodegenerative vulnerability within PDD-linked conditions carries important implications. Adults with ASD, particularly those with coexisting intellectual disability, epilepsy, or genetic syndromes, appear to experience higher rates of premature mortality and may show atypical cognitive aging [2]. However, this area remains underexplored. Most longitudinal studies end in early adulthood, leaving a significant gap in understanding the neurobiological trajectory across the lifespan. As life expectancy increases for individuals with neurodevelopmental disorders, research must shift to include mid-life and older adults, examining risks for dementia, movement disorders, or accelerated neurobiological aging.

Another domain requiring attention is the role of chronic stress, sensory dysregulation, and psychiatric comorbidities in modifying long-term neural health. Anxiety, depression, and chronic stress, which are prevalent in ASD, are known contributors to neuroinflammatory and neurodegenerative processes in the general population. Whether these factors accentuate neural vulnerability in PDD-related conditions is an open and clinically relevant question [2].

From a translational perspective, the potential overlap between neurodevelopmental and neurodegenerative biology offers opportunities for shared therapeutic strategies. Early interventions targeting synaptic stabilization, mitochondrial optimization, or neuroimmune modulation may have long-term neuroprotective benefits. At the same time, biomarker research, spanning neuroimaging, inflammatory markers, and genetics, may help identify individuals at risk for atypical neurological aging [3].

Ultimately, revisiting the concept of neurodegeneration within the spectrum of PDD is not an attempt to redefine ASD or related conditions as degenerative [2]. Rather, it is a call to refine our understanding of brain vulnerability across the lifespan. As the boundaries between developmental and degenerative neuroscience become increasingly porous, clinicians, researchers, and policymakers must adopt an integrated framework that captures both early-life neurodevelopment and long-term neural health.

The time has come to bridge the conceptual gap between development and degeneration. A lifespan-oriented model, grounded in biology, attentive to clinical outcomes, and informed by patient experience, may ultimately yield a more comprehensive and humane understanding of individuals previously identified within the PDD spectrum.
